# Shade Tree Selection in Cocoa Agroforestry: Ghanaian Farmers' Preferences, Ecological Insight and Drivers of Local Ecological Knowledge

**DOI:** 10.1002/ece3.71685

**Published:** 2025-06-30

**Authors:** Michael Asigbaase, Solomon Batamia Ndego, Belinda Effah

**Affiliations:** ^1^ Department of Forest Sciences University of Energy and Natural Resources Sunyani Ghana

**Keywords:** adoption barriers, conservation, ecosystem services, IUCN red list, local ecological knowledge, social‐ecological systems

## Abstract

Integrating shade trees into cocoa farms potentially reduces the environmental cost of cocoa production and enhances their conservation value. However, it is unclear how farmers' shade trees preferences vary across cocoa production stage and how these preferences influence biodiversity conservation outcomes, including tree species at risk. Therefore, grounded in the Social‐Ecological Systems framework, we collected data from 363 cocoa farmers via questionnaire‐led interviews and farmer responses regarding shade tree preferences and knowledge using linear mixed‐effects models, cluster analysis, and mean rating scores. The results showed that farmers' local ecological knowledge was primarily influenced by membership in farmer‐based organizations, number of information sources, credit access frequency, and cocoa production stage. Farmers cited 23 preferred shade tree species, indicating a moderate pool of preferred shade tree species among cocoa farmers. *Albizia ferruginea* and *Newbouldia laevis* were uniquely preferred by farmers with young cocoa farms while *Senna siamea* was unique to old cocoa farmers. Jaccard dissimilarity indices indicated that species composition became increasingly distinct as cocoa plantations aged (27.3% dissimilarity in younger farms vs. 65% in older farms), yet overall shade tree diversity remained stable. Overall, 40%–57% of preferred shade trees required conservation priority based on International Union for Conservation of Nature Red List and national star rating. It was concluded that socio‐economic factors including information access, and institutional support mediate conservation‐oriented behavior in agroforestry landscapes and that the composition of farmers' preferred shade tree species changes as the plantation ages, but with a stable diversity. These findings suggest that integrating farmers' preferred shade trees on cocoa farms has a potential tree species conservation value. The study extends Social‐Ecological Systems applications by highlighting how system components interact to influence conservation behavior. Investigating the long‐term impacts of shade tree diversity on cocoa agroforestry farms is critical to enhance their integration and cocoa productivity.

## Graphical abstract

The study found a moderate (23) pool of preferred shade tree species among cocoa farmers, with specific species favored at different cocoa production stages, which highlights the need for diversified agroforestry systems to enhance resilience and sustainability. Farmers varied shade tree species composition as the cocoa plantation ages, but not their diversity per se. They prioritized shade tree species with characteristics (e.g., fast‐growth, small crowns, and evergreen foliage) that maximize cocoa farm productivity while minimizing competition for resources; thus, balancing ecological and economic benefits.

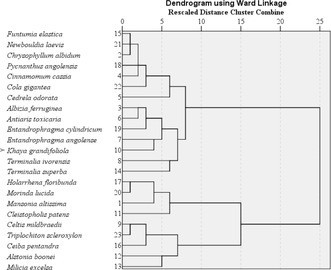

## 1 Introduction

Cocoa agroforestry systems have the potential to balance ecological, economic, and social benefits in tropical regions (Tscharntke et al. 2011; Vaast and Somarriba 2014). Central to these systems is the integration of shade trees, which provide critical ecological services, including shade for cocoa, biodiversity enhancement, soil fertility improvement, carbon sequestration, and microclimate regulation (Dawoe et al. 2016; Asigbaase et al. 2019; Vaast and Somarriba 2014; Asare et al. 2014). Additionally, it offers opportunities, such as food production, risk reduction through diversification, mitigation of deforestation and soil degradation (Schroth and Ruf 2014; Somarriba et al. 2021). The extensive root systems of shade trees prevent soil erosion, particularly in regions with heavy rainfall, while leaf litter acts as natural mulch, enriching soil fertility and promoting the growth of beneficial soil microbes (Tscharntke et al. 2011; Wessel and Quist‐Wessel 2015). These benefits are particularly pertinent in West Africa, which produces over 70% of the world's cocoa, where smallholder farmers dominate the sector and face challenges, such as declining soil fertility, pest and disease pressures, and climate variability (Wessel and Quist‐Wessel 2015; Dawoe et al. 2016; Asare et al. 2014). For Ghana, cocoa production is a vital economic activity, accounting for about 30% of export earnings and contributing around 29% to the country's GDP (Duguma et al. 2001; Boansi 2013; Ajagun et al. 2022). That notwithstanding, the success of cocoa agroforestry depends significantly on farmers' preferences for and management of shade trees (Asare 2005; Anglaaere et al. 2011; Asigbaase 2019). However, there is limited understanding of how these preferences vary across different cocoa production stages (i.e., from young to mature cocoa plantations) and influence conservation outcomes (Asigbaase et al. 2019; Dawoe et al. 2016), which limits the design of agroforestry systems that align with both scientific and local practices. Moreover, the selection and management of shade trees remain deeply influenced by farmers' local ecological knowledge (LEK) and preferences, which are shaped by dynamic interactions between cultural, ecological, and socioeconomic factors.

This study is grounded in the Social‐Ecological Systems (SES) framework, which provides a holistic lens to explore the interconnectedness of human and ecological systems (Ostrom 2009). The SES framework emphasizes the dynamic feedback loops between social systems, such as farmer communities, and ecological systems, such as cocoa agroforestry landscapes. Within this framework, shade trees in cocoa systems can be understood as pivotal components of ecological subsystems that interact with farmers' knowledge, decisions, and cultural norms. By examining these interactions, the SES framework facilitates a nuanced understanding of how farmers' preferences and LEK influence, and are influenced by, ecological processes and external drivers, such as market forces or climate change.

Farmers' preferences for shade trees are often informed by their understanding of the multifunctional roles these trees play. Although some farmers prioritize tree species that offer economic benefits, such as timber or non‐timber forest products, others emphasize ecological functions, such as improving soil health or providing natural pest control (Graefe et al. 2017; Esche et al. 2023). These preferences possibly vary across the production stages of cocoa farms, reflecting evolving needs and priorities as cocoa trees mature. Although previous studies have examined farmers' preferences for shade trees in cocoa agroforestry systems (Asare 2005; Anglaaere et al. 2011), there remains a limited understanding of how these preferences evolve across different cocoa production stages and their implications for tree conservation and agroforestry sustainability. Cocoa farmers manage shade trees to meet the ecological requirements of cocoa trees and their own economic or domestic needs (Asigbaase et al. 2019). Understanding how farmers' choices align with broader conservation goals is crucial for bridging gaps between local practices and global sustainability efforts. Equally important is the convergence or divergence between farmers' LEK and mainstream ecological knowledge (MEK) regarding the ecosystem services and disservices of shade trees. Although LEK is rooted in experiential and cultural insights, MEK is often derived from scientific research and ecological modeling. Discrepancies between these knowledge systems can lead to mismatches in agroforestry interventions, undermining their effectiveness (Carpente 2020). Conversely, identifying areas of convergence can inform participatory approaches to agroforestry management, fostering practices that are ecologically sound and locally relevant.

The drivers of LEK about shade trees on cocoa farms are equally complex, encompassing factors, such as access to education, market dynamics, social networks, and exposure to extension services (Fleming et al. 2019; Ahmad et al. 2023). These drivers influence not only the knowledge farmers hold but also their capacity to adapt and innovate in response to changing ecological and economic conditions. A nuanced understanding of these drivers is essential for designing context‐specific interventions that empower farmers and enhance the sustainability of cocoa agroforestry systems.

The objectives of the study were: (i) to evaluate the dynamics, composition and diversity of farmers' preferences for shade trees in cocoa agroforestry systems along a production stage gradient and explore how these preferences impact conservation of tree species; (ii) to analyze farmers' priority ecosystem services across different cocoa production stages; (iii) to examine the convergence, divergence and knowledge gaps between local ecological knowledge about the ecosystem (dis)services of shade trees on cocoa farms and mainstream ecological knowledge; (iv) to analyze the drivers of farmers' local ecological knowledge of shade trees on cocoa farms; and (v) to explore perceived opportunities and barriers to shade tree integration on cocoa farms.

Our study provides essential data and insights that can inform local, regional, and global strategies for enhancing the sustainability and resilience of agroforestry systems, particularly in cocoa production, thus contributing to broader development and environmental objectives. For example, it aligns with the African Union's Agenda 2063, which stresses the need for sustainable agricultural practices to ensure food security, biodiversity conservation, and climate resilience in Africa (African Union 2015). At the global scale, the study contributes to the achievement of the United Nations Sustainable Development Goals (SDGs), particularly SDG 2 (Zero Hunger), SDG 13 (Climate Action), and SDG 15 (Life on Land). Moreover, understanding local ecological knowledge helps bridge the gap between traditional knowledge and modern environmental science, fostering inclusive and sustainable agricultural practices (Shilomboleni et al. 2024).

## 2 Methodology

### 2.1 Study Description

**FIGURE 1:**
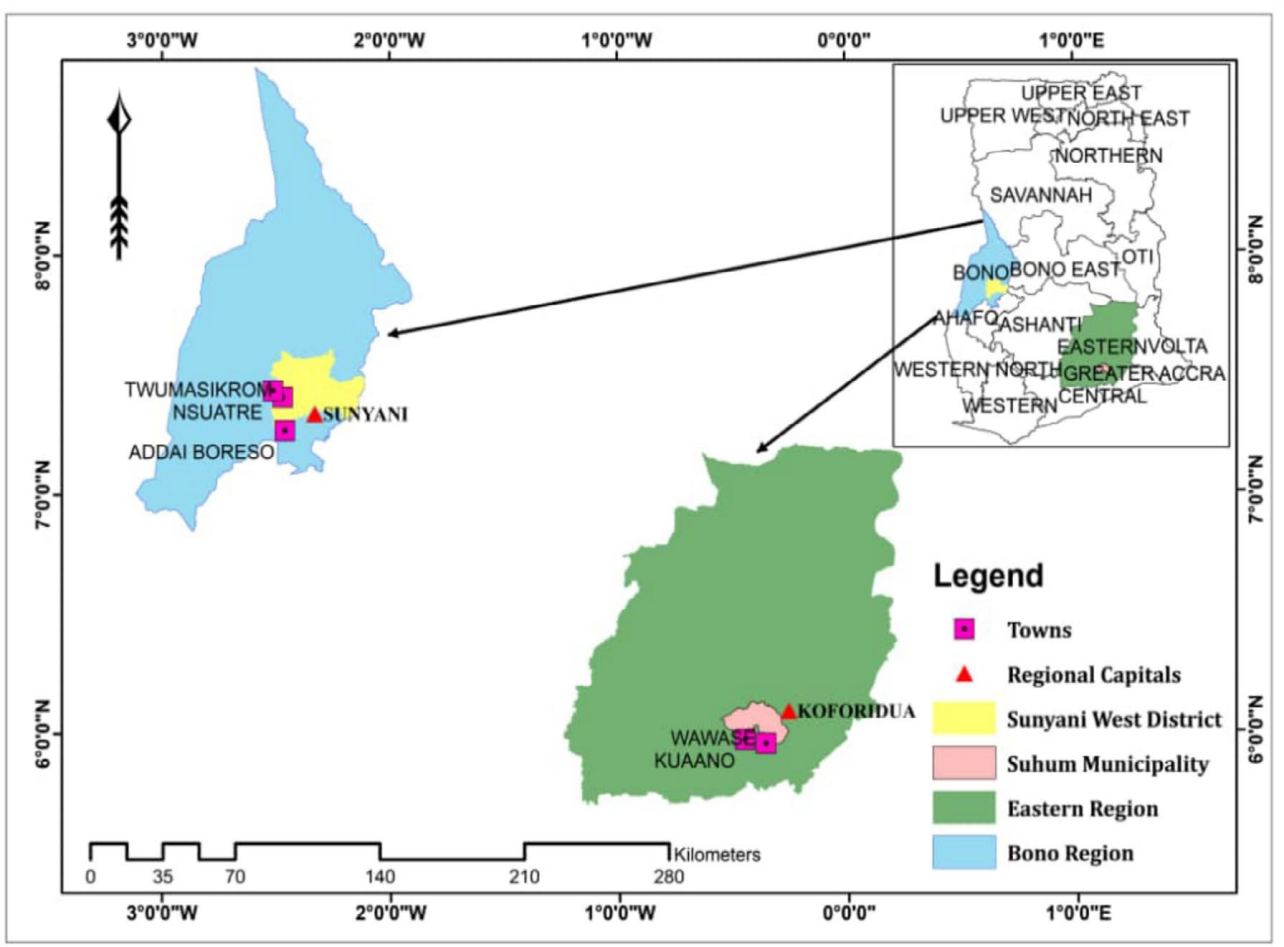
Study area map showing study districts, towns and regional capitals.

The study took place in Suhum and Sunyani West Municipalities (Figure 1). Suhum Municipality spans 400 km^2^ and is bordered by New Juaben Municipality to the northeast, East Akim to the north, Ayensuano District to the west and south, and Akwapim North Municipality to the east. The municipality's population is approximately 90,358, with a gender distribution of 48.7% males and 51.3% females (Ghana Statistical Service (GSS) 2014, 2021). Situated in Ghana's tropical zone (moist semi‐deciduous forest zone), the area experiences temperatures ranging from 24°C to 29°C and receives annual rainfall between 1270 mm and 1651 mm. The district's fertile soil and favorable climate make agriculture the dominant economic activity, with about 57.8% of households engaged in farming (Ghana Statistical Service (GSS) 2014, 2021). Key crops include *Dioscorea* spp, *Manihot esculenta, Pennisetum glaucum, Sorghum bicolor, Vigna unguiculata, Oryza sativa, Arachis hypogaea*, and *Citrullus lanatus* , alongside cash crops like *Anacardium occidentale, Mangifera indica*, and *Nicotiana tabacum* . Vegetable farming, particularly *Solanum lycopersicum* and *Solanum aethiopicum* , is also prevalent (Ghana Statistical Service (GSS) 2014).

The Sunyani West Municipality (SWM) is situated between latitudes 7° 19′ N and 7° 35′ N and longitudes 2° 08′ W and 2° 31′ W. The Municipal shares borders with Wenchi Municipal to the north, Offinso North to the east, Sunyani Municipal to the south, Berekum Municipal to the west, Dormaa Municipal and Dormaa East to the southwest, and Tain District to the northwest. Covering 1059.33 km^2^, it represents 4.2% of the region's total land area (GSS 2014, 2021). The Sunyani West Municipality has a population of 136,022, with a gender distribution of 49.4% males and 50.6% females (GSS 2021). Located in Ghana's tropical zone, the area experiences average temperatures ranging from 25°C to 30°C and receives annual rainfall of approximately 1073 mm to 1700 mm. Agriculture is the predominant economic activity, employing about 60% of households, with 96% of these engaged in crop farming. This climate supports moist semi‐deciduous forest vegetation, including two main forest reserves: Tain I and II and the Yaya Forest Reserves. Common timber species in these reserves include *Milicia excelsa* (Welw.) C.C.Berg, *Khaya ivorensis* A.Chev., *Triplochiton scleroxylon* K.Schum., *Terminalia superba* Engl. & Diels, *Tectona grandis* L.f., *Antiaris toxicaria* (Pers.) Lesch., *Entandrophragma cylindricum* (Sprague) Sprague, and *Ceiba pentandra* (L.) Gaertn. The Municipal also features secondary vegetation for agricultural and other uses. The terrain is generally undulating, ranging from 213.36 m along the River Bisi Basin to 335.28 m near Chiraa, with a dendritic drainage pattern. The Tano River is a key water source for domestic and agricultural use during dry seasons. Other significant rivers include the Abisu, Sise, Nyinahini, Ahunyan, Bisi, and Bore.

### 2.2 Research Design and Methods

We adopted a mixed‐methods design that explicitly aligns qualitative and quantitative components with our objectives. For evaluating farmers' preferences for shade trees along a production‐stage gradient and examining convergence/divergence between local ecological knowledge (LEK) and mainstream ecological knowledge (MEK), semi‐structured interviews capture nuanced criteria for species selection, conservation implications, and LEK versus MEK (Enosh et al. 2015; Creswell and Plano Clark 2017). To analyze the drivers of farmers' ecological knowledge, a structured questionnaire administered to the cocoa farmers yielded generalizable data on socioeconomic, farm and demographic predictors (Appendices S2 and S3) (Bryman 2016; Kouassi et al. 2021; Doe et al. 2023). Finally, exploring perceived opportunities and barriers to shade‐tree integration leverages semi‐structured questionnaires for depth and survey data for prevalence and ranking of barriers (Dawadi et al. 2021).

### 2.3 Study Population and Sample Size Determination

In this study, the target population was cocoa farmers within the study area (Figure 1). We calculated the target sample size (363) for our farmer surveys using Yamane's approach (Equation (1); Yamane 1967).

(1) $$n=\frac{N}{\left(1+N*e^{2}\right)}$$

where ‘*n*’ is the sample size, ‘*N*’ is the population size, and ‘*e*’ is the desired margin of error (0.05).

### 2.4 Data Collection Approach

**FIGURE 2:**
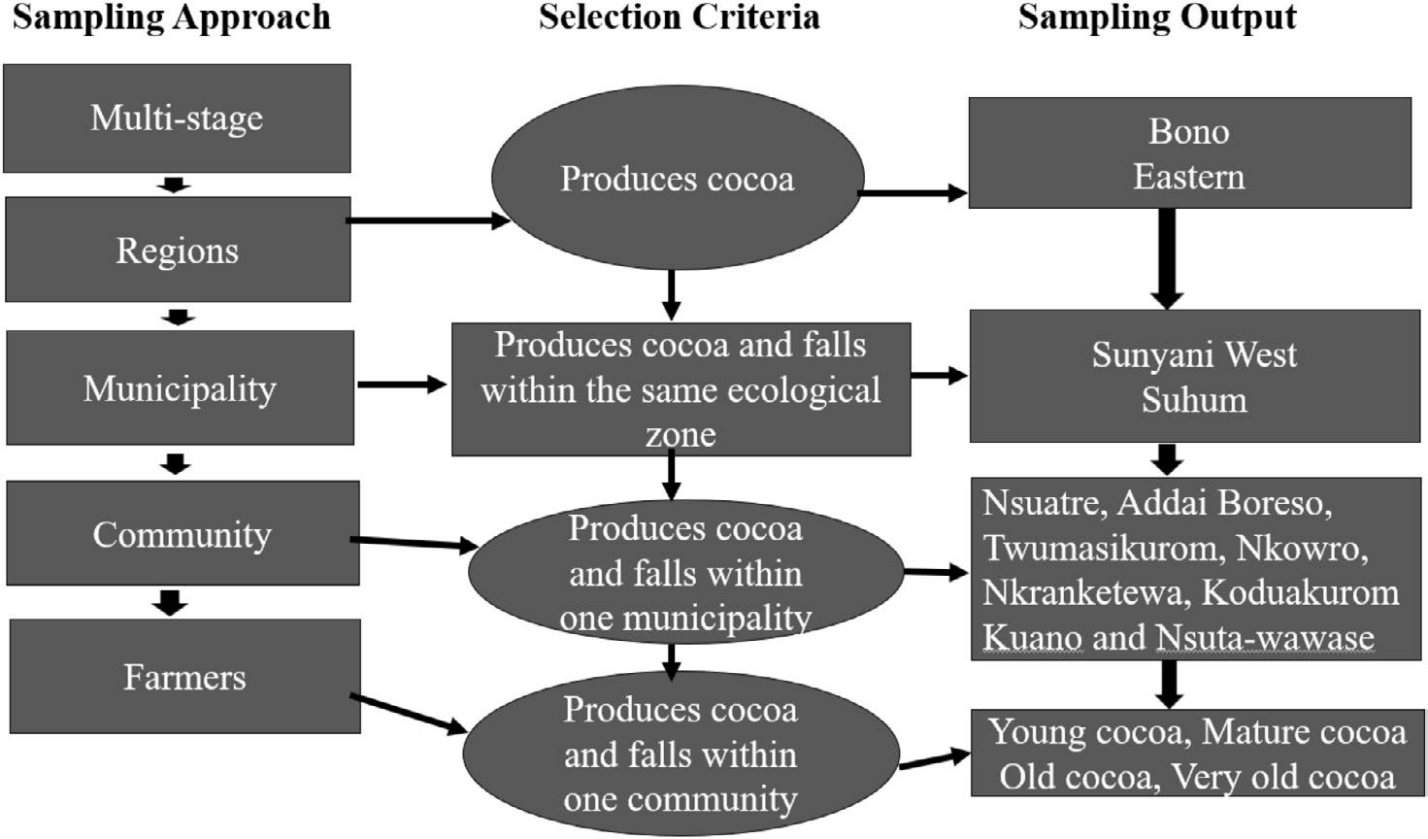
A flowchart of sampling approach, selection criteria, and output.

We used a four‐step multi‐stage sampling approach to select study regions, municipalities, communities, and cocoa farmers (Figure 2). Specifically, two cocoa producing regions were randomly selected from a list of cocoa producing regions in Ghana. After this, one municipality in each region was randomly selected based on the availability of cocoa farmers to participate in the research. Next, six cocoa growing communities (Nsuatre, Addai Boreso, Twumasikurom, Nkowro, Nkranketewa, and Koduakurom) in Sunyani West Municipality and two communities (Kuano and Nsuta‐Wawase) in the Suhum Municipality were randomly selected for the study. In each selected community, cocoa farmers (363) were randomly selected from a list of cocoa farmers in each community provided by the local offices of COCOBOD, farmer groups, and licensed buying companies (LBCs). Prior to this, the cocoa farms were grouped into four categories: young cocoa farms (< 6 years), mature cocoa farms (6–25 years), old cocoa farms (26–45 years), and very old cocoa farms (> 45 years), reflecting cocoa production phases. The required data was gathered through the use of a questionnaire‐led interviews (1–3 h), from March to June 2024. The questionnaire (Appendix S1) was structured into four key sections: (i) demographic and farm characteristics (summarized in Appendix S2), (ii) shade tree preferences across cocoa production stages, (iii) farmers' perceptions of ecosystem services and disservices, and (iv) barriers and opportunities for shade tree integration. Questions were designed using a mix of closed‐ended, Likert‐scale, and open‐ended formats to capture both quantitative and qualitative insights. Pre‐testing was conducted with 30 farmers to refine clarity and cultural appropriateness. Additionally, expert consultations with three agroforestry researchers and two extension officers ensured content relevance and alignment with scientific perspectives. The interviews were conducted at the farms of the farmers while they were resting. We verified the presence of preferred trees on their farms. Our study adhered to institutional (Department of Forest Science Ethics Committee, University of Energy and Natural Resources) and international guidelines (Helsinki Declaration of 1975, as revised in 2008). Participation in the research was voluntary, and informed consent was obtained from participants before they were engaged.

### 2.5 Data Processing and Analysis

The collected data from the questionnaires and household interviews were entered into a Microsoft Excel spreadsheet. After organizing the data in Excel, further analysis was conducted using Statistical Package for Social Science (SPSS) version 25 for Windows. The composition of preferred shade trees across the cocoa production was analyzed using Jaccard's similarity index (Equation (2)) and Jaccard's distance (Equation (3)) and Shannon diversity index was estimated for each cocoa production stage and relative Shannon diversity index was estimated as the difference between two cocoa production stages expressed as a percentage. The conservation status of preferred shade trees was assessed using IUCN Red List and the Star Rating System in Ghana (Hawthorne 1995). Ghana's conservation framework assigns species to five “star” categories: Black Star for taxa that are globally rare and nationally uncommon; Gold Star for those relatively rare on a global or national scale; Blue Star for species rare in one context but common in the other; Scarlet Star for nationally common taxa under intense exploitation; and Red Star for widespread species facing significant harvest pressure—each category guiding tailored management and protection measures (Hawthorne 1995). The LEK‐derived ecological traits and services, and uses of preferred shade trees were compared with MEK obtained through literature review (e.g., Plant Resources of Tropical Africa, Useful Plants of Tropical West Africa and Agroforestree databases) to identify areas of convergence (alignment between farmers' perceptions and scientific knowledge), divergence (differences in perceptions vs. scientific understanding), and knowledge gaps (aspects covered by LEK but with insufficient scientific validation or vice versa) of the ecosystem (dis)services of shade trees on cocoa farms. This was done using descriptive statistics to highlight key patterns. Additionally, hierarchical cluster analysis with ward's linkage and Euclidean distance metric was conducted to analyze the local ecological traits and services of shade trees (LEK dataset). The results were visualized in a dendrogram, with the *x*‐axis showing rescaled distance (dissimilarity) and the *y*‐axis listing shade tree species. Distinct clusters suggest that farmers differentiate between groups of shade trees, based on their perceived ecological traits and services or uses. To determine priority ecosystem services across the production gradient, mean Likert‐scale [1 (lowest) to 5 (highest)] rating scores were used. Similarly, to evaluate perceived barriers and opportunities for shade tree integration, Likert‐scale [1 (lowest) to 5 (highest)] rating scores and One‐Way ANOVA with Fisher's Least Significant Difference pairwise comparisons were conducted. Local ecological knowledge was estimated as the sum of the scores reflecting a farmer's ability to cite shade tree species, their uses, and expression of facts of the ecological benefits or disbenefits of shade trees. A score of one (1) was awarded for each citation or factual expression. Thus, farmers who cited more shade trees and could identify them, specific uses for each cited shade tree and expressed more factual statements demonstrated greater local ecological knowledge. Linear Mixed‐Effects Model was employed to analyze the fourth objective, which focused on the drivers of farmers' local ecological knowledge of shade trees on cocoa farms. Mixed linear regression was chosen due to its capability to handle both fixed and random effects of the data collected. The regression included socio‐demographic, farm, and economic attributes as fixed factors with study region as the random factor. The predictors included gender, marital status, origin, religion, household size, number of years of residing in the community, respondent age, number of household adults (≥ 18 years), number of household children (< 18 years), number of farmlands, total size of farmlands, cocoa farming experience, major season cocoa yield, minor season cocoa yield, mean monthly cocoa income, mean monthly non‐cocoa income, frequency of extension services, number of information sources, and number of farmer‐based organizations a farmer belongs (Appendices S2 and S3). Model selection involved comparing models with different combinations of independent variables using the Akaike Information Criterion (AIC) to identify the best‐fitting model (Akaike 1973; Burnham and Anderson 2002). Models were compared based on the AIC, with the model having the lowest AIC selected as the best‐fitting model. Additionally, likelihood ratio tests were used to compare nested models. Model validation involved checking the assumptions and assessing the goodness‐of‐fit. Residual diagnostics were performed to check for normality, homoscedasticity, and independence of residuals.

(2) $$J=\left(\frac{c}{a+b+c}\right)*100$$

(3) JD=1−J

Where *J* is Jaccard's similarity index, *JD* is Jaccard's distance, ‘*c*’ denotes the number of preferred shade tree species common to two different cocoa production stages, ‘*a*’ and ‘*b*’ denotes the number of preferred shade tree species unique to the different cocoa production stages. Higher *J* and lower *JD* values indicate high similarity (i.e., farmers prefer largely the same tree species across stages) and vice versa.

## 3 Results and Discussion

### 3.1 Dynamics of Farmers' Shade Tree Preferences Across Different Cocoa Production Stages

Asigbaase et al. (2019) reported 27–41 shade tree species in different aged cocoa farms in Ghana while Dawoe et al. (2016) reported 34–49 shade trees. Our farmers cited a total of 23 preferred shade tree species, indicating a moderately diverse yet selective pool of species, likely shaped by familiarity, ecological benefits, and functional value (Table 1; Sonwa et al. 2007; Amoako and Asare 2015). Notably, *Cedrela odorata* , *Terminalia ivorensis* , *Khaya grandifoliola*, and *Terminalia superba* consistently ranked among the top preferred species across all cocoa production stages. This consistency reflects farmers' prioritization of multifunctional species known to offer high‐quality shade, improve soil fertility, and support cocoa productivity (Asare 2005; Gockowski and Sonwa 2011; Asare et al. 2014). These findings suggest that farmers' preferences are not arbitrary but grounded in accumulated experiential knowledge and ecological reasoning that align with agronomic outcomes and long‐term sustainability (Asare 2005; Anglaaere et al. 2011). From an SES perspective, this indicates a form of adaptive knowledge application, wherein farmers integrate species that simultaneously meet livelihood needs and support ecological resilience.

**TABLE 1:** Cited preferred shade trees across different cocoa age stages and their conservation status.

Local name	Scientific name	IUCN status	Family	Species guild	Star rating	Population trend	Citation frequency			
							YCF	MCF	OCF	VOCF
Watapuo	*Cola gigantea* A. Chev.	Least Concern	Malvaceae	NPLD	Green	Unspecified	2	1	0	0
Cassia	*Senna siamea* (Lam.) H.S. Irwin & Barneby	Least Concern	Fabaceae	Pioneer	—	Decreasing	0	0	1	0
Cedrela	*Cedrela odorata* L.	Vulnerable	Meliaceae	Pioneer	—	Decreasing	36	58	25	15
Chenchen	*Antiaris toxicaria* Lesch.	Least Concern	Moraceae	NPLD	Red	Stable	1	0	0	1
Edinam	*Entandrophragma angolense* Welw. C.DC.	Near Threatened	Meliaceae	NPLD	Red	Decreasing	2	6	2	0
Emire	*Terminalia ivorensis* A.Chev.	Vulnerable	Combretaceae	Pioneer	Scarlet	Unspecified	51	105	60	40
Esa	*Celtis mildbraedii* Engl.	Least Concern	Cannabaceae	Shade‐bearer	—	Unspecified	0	3	1	0
Konkroma	*Morinda lucida* Benth	Least Concern	Rubiaceae	Pioneer	Green	Stable	8	39	18	0
*Mahogany*	*Khaya grandifoliola C.D.C*.	Vulnerable	Meliaceae	NPLD	Red	Unspecified	43	63	30	25
Oprono	*Mansonia altissima* (A.Chev.) A. Chev.	Least Concern	Malvaceae	NPLD	Pink	Stable	2	2	0	0
Odum	*Milicia excelsa* (Welw.) C.C.Berg.	Near threatened	Moraceae	Pioneer	Scarlet	—	8	34	8	3
Ofram	*Terminalia superba* Engl. & Diels.	Vulnerable	Combretaceae	Pioneer	Scarlet	—	69	106	61	41
Ofuntum	*Funtumia elastica* (Preuss) Stapf	Least Concern	Apocynaceae	NPLD	Pink	Stable	1	1	0	0
Onyina	*Ceiba pentandra* (Linn.) Gaertn.	Least Concern	Malvaceae	Pioneer	Green	Unspecified	2	4	0	1
Otie	*Pycnanthus angolensis* (Welw.) Warb.	Least Concern	Myristicaceae	NPLD	Red	Stable	1	3	0	0
Abe	*Elaeis guineensis* Jacq.	Least Concern	Arecaceae	Pioneer	Pink	Unspecified	1	0	0	0
Sapele	*Entandrophragma cylindricum* (Sprague)	Vulnerable	Meliaceae	NPLD	Scarlet	Stable	0	1	1	0
Sese	*Holarrhena floribunda* (G.Don) T.Durand & Schinz	Least Concern	Apocynaceae	Pioneer	Green	Stable	5	9	10	0
Nyame dua/Sinuro	*Alstonia boonei* De Wild.	Least Concern	Apocynaceae	Pioneer	Green	Decreasing	5	16	9	0
Susumasa	*Newbouldia laevis* (P.Beauv.) Seem.	Least Concern	Bignoniaceae	Pioneer	Green	Stable	1	0	0	0
Wawa	*Triplochiton scleroxylon* K.Schum.	Least Concern	Malvaceae	Pioneer	Scarlet	Unspecified	2	19	1	0
Awiemfuosemina	*Albizia ferruginea* (Guill. & Perr.) Benth.	Near Threatened	Fabaceae	NPLD	Scarlet	Decreasing	2	0	0	0

Abbreviations: MCF, Mature cocoa farms; NPLD, Non‐pioneer light demander; OCF, Old cocoa farms; VOCF, Very old cocoa farms; YCF, Young cocoa farms.

Furthermore, variation in shade tree preferences across cocoa production stages reflects farmers' responsiveness to the evolving biophysical and management demands of their systems. For instance, *Milicia excelsa* was commonly preferred during the young and very old stages, possibly due to its strong shade and timber value at early and declining productivity phases, whereas *Albizia ferruginea* —a nitrogen‐fixer—was preferred during the young stage, when soil enrichment is critical (Orwa et al. 2009; Jannah et al. 2022). Meanwhile, *Senna siamea* appeared more frequently in older cocoa farms, potentially reflecting a shift toward trees with more durable structural properties or lesser competitive pressure at later stages. These dynamic patterns of preference underscore the strategic nature of farmers' shade tree selection, tailored to the developmental needs of cocoa and broader livelihood considerations. Moreover, the shifts in preferences align with the SES framework's emphasis on feedback mechanisms, as ecological conditions and management practices evolve alongside cocoa systems (Asigbaase et al. 2019; Carpente 2020).

These findings illustrate how farmer preferences are not static but evolve in response to production‐stage‐specific requirements. They also suggest that promoting species aligned with farmers' knowledge and ecological rationale could enhance productivity outcomes.

### 3.2 Composition and Diversity of Farmers' Preferred Shade Trees Species Across Different Cocoa Production Stages

The composition of preferred shade tree species tended to be increasingly dissimilar as the cocoa plantation ages, suggesting that farmers possibly adapt their selection criteria to address shifting ecological and economic priorities, such as provision of shade, food and fruits and timber (Asigbaase et al. 2019). For example, the composition of preferred shade tree species by young and mature cocoa farmers was 72.7% similar while that of young and very old cocoa farmers was 35.0%. Again, mature and old cocoa farms were composed of similar preferred shade trees (68.4%) while mature and very old cocoa farms were composed of dissimilar preferred shade trees. This pattern indicates a shift in farmers' preferred tree species as cocoa plantations age, with farmers managing older cocoa plantations tending to favor a different set of shade trees compared to those managing younger ones (Asigbaase et al. 2019; Carpente 2020). Additionally, given that mature and very old cocoa plantations showed even greater dissimilarity in preferred shade tree species suggests a gradual evolution in the selection of shade tree species as the cocoa plantation age (Asigbaase et al. 2019). Therefore, the observed pattern may be an adaptive response to the co‐evolution of social and ecological subsystems.

We found that even though the dissimilarity of preferred shade trees tended to increase with the cocoa systems age, the diversity of preferred shade trees tendered to be relatively stable for young, mature and old cocoa farmers. However, the diversity of preferred shade trees by farmers when the cocoa trees are very old was relatively lower compared to the young, mature and old cocoa stages (Table 2), possibly reflecting changing ecological requirements and management practices as the cocoa plantation ages (Asigbaase et al. 2019) or economic demands (Aneani et al. 2012). Boadi et al. (2024) documented similar patterns of declining plant diversity in aging agroforestry systems. In general, cocoa trees initially require shade to establish, but farmers actively manage the trees when the cocoa systems mature to enhance cocoa productivity (Asare 2005; Dawoe et al. 2016; Asigbaase et al. 2019) and this may explain the documented pattern.

**TABLE 2:** Composition and relative diversity of preferred shade trees across different cocoa production age stages.

	Jaccard similarity index (%)	Jaccard distance (%)	Relative Shannon diversity index (%)
YCF vs. MCF	72.7	27.3	6.4
YCF vs. OCF	50.0	50.0	3.0
YCF vs. VOCF	35.0	65.0	29.1
MCF vs. OCF	68.4	31.6	9.2
MCF vs. VOCF	31.6	68.4	33.7
OCF vs. VOCF	31.2	68.8	26.9

Abbreviations: MCF, Mature cocoa farms; OCF, Old cocoa farms; VOCF, Very old cocoa farms; YCF, Young cocoa farms.

### 3.3 Implications of Farmers' Shade Tree Preferences on Tree Species Conservation and Cocoa Production

Our results demonstrates that all the preferred shade tree species, except *Cedrela odorata* and *Senna siamea* , are native species (Table 1). This overwhelming preference for native species suggests that farmers value trees that are well‐adapted to local ecological conditions and have co‐evolved with native biodiversity. Ecologically, the predominance of native species is significant, as they tend to support richer assemblages of forest‐dependent taxa, including birds, bats, insects, and fungi, thereby enhancing the conservation potential of cocoa agroforestry systems (Daghela Bisseleua et al. 2013; Ocampo‐Ariza et al. 2023).

**FIGURE 3:**
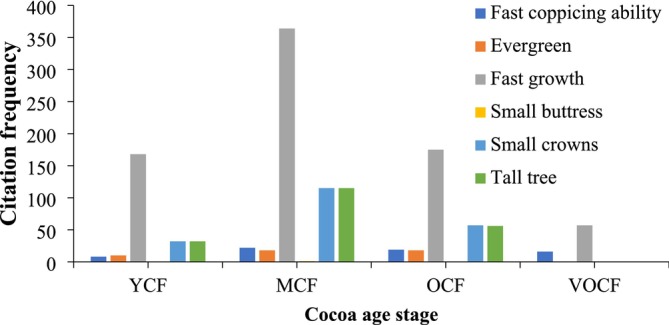
Qualities of preferred shade trees across cocoa age stages. Citation frequency is the number of mentions by farmers in structured surveys (*n* = 363) when asked why they prefer specific shade trees. MCF = Mature cocoa farms; OCF = Old cocoa farms; VOCF = Very old cocoa farms; YCF = Young cocoa farms.

Furthermore, given that 40%–57% of the cited preferred shade tree species require conservation priority based on the IUCN Red List and local star ratings, integration of preferred shade trees may contribute significantly to on‐farm biodiversity conservation (Table 1; Asigbaase et al. 2019; Asigbaase et al. 2023). This convergence between local preferences and conservation priorities underscores the potential of cocoa agroforestry systems to function as de facto conservation landscapes, given that farmers preferred diverse shade trees (Boadi et al. 2024). From a Social‐Ecological Systems (SES) perspective, the convergence between local practices and potential conservation outcomes reflects the SES framework's assertion that socioecological resilience emerges from the interactions between human agency and ecological processes. The consistency of farmer preferences across different cocoa production stages—favoring pioneer or non‐pioneer light demanders that are tall, fast‐growing, small‐crowned, evergreen, fast‐coppicing, and possess small buttresses—points to a selection logic grounded in the optimization of both ecological and agronomic functions (Figure 3; Anglaaere et al. 2011; Asigbaase et al. 2019). Such traits enhance canopy structure and reduce interspecific competition for light and space, maintain favorable microclimatic conditions, and enable easier management of tree‐crop interactions, providing a balance between shade provision and cocoa cultivation needs (Asare 2005; Anglaaere et al. 2011; Asigbaase et al. 2019).

In addition to these, farmers preferred trees with qualities, such as fast coppicing ability, small buttresses and evergreen, reflecting a preference for certain functional traits (Boadi et al. 2024; Asigbaase et al. 2019). Trees with small buttresses reduce competition for root space with cocoa roots and facilitate easier management, whereas those with evergreen foliage ensures continuous shade and ecological stability throughout the year (Anglaaere et al. 2011; Asigbaase 2019). Furthermore, trees with fast‐coppicing ability can easily regenerate and be managed for continuous leaf cover and quick recovery after pruning or harvesting (Jagoret et al. 2014). Farmers also consider the practical applications of shade trees, including timber, fuelwood, medicinal uses, and construction materials. This multifunctionality supports the integration of shade trees into cocoa systems, as it contributes to the resilience and sustainability of cocoa farms (Anglaaere et al. 2011; Sonwa et al. 2014). By integrating species that offer multiple benefits, farmers can enhance the ecological and economic viability of their agroforestry systems (Asare et al. 2014). This reflects the SES framework's emphasis on the importance of resource users' knowledge in fostering adaptive management strategies that sustain both livelihoods and ecosystem functions.

### 3.4 Farmers' Local Knowledge of the Ecological Traits and Services of Shade Trees on Cocoa Farms

**FIGURE 4:**
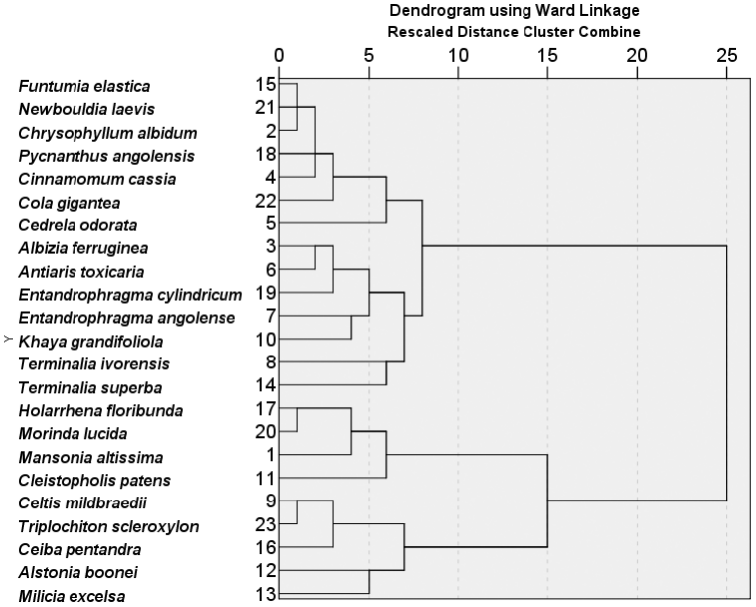
Dendrogram from hierarchical cluster analysis of ecological traits and services of preferred shade trees. Trees merging at lower distances share similar ecological traits and services, whereas those at higher distances reflect distinct ecological traits and services.

The study identified a wide variety of shade tree species preferred by farmers, reflecting their in‐depth local ecological knowledge and their understanding of the ecological traits and services provided by shade tree species (Table 3). The hierarchical cluster analysis using Ward's linkage method revealed four distinct clusters of preferred shade tree species based on similarities in ecological traits and services, as well as species uses (Figure 4). Cluster one composed of *Funtumia elastica, Newbouldia laevis, Chrysophyllum albidum, Pycnanthus angolensis, Senna siamea, Cola gigantea*, and *Cedrela odorata* . This cluster included species known for providing a variety of uses, such as woodwork, medicinal purposes, and fuelwood. Ecological traits like fast growth, quality shade, and soil fertility improvement were common, suggesting that the preference for species in cluster one was driven by their ability to provide quick returns in terms of biomass and utility, which is crucial for farmers looking to maximize productivity and economic benefits (Lemmens et al. 2012; Ashton et al. 2014). *Cedrela odorata* is valued for its deep roots and soil‐enriching properties, which enhance cocoa yields by reducing erosion and nutrient leaching (Piotto et al. 2004; Orwa et al. 2009). Species that enhance soil nutrients, structure, and organic matter are essential for maintaining soil health and supporting cocoa productivity (Kaba et al. 2020; Asitoakor et al. 2024). Farmers' preference for shade trees known to improve yields and microclimatic conditions reinforces earlier findings on the positive role of shade in cocoa systems (Asare 2005; Asigbaase 2019; Anglaaere et al. 2011). Notably, the selection of fast‐growing, pest‐resistant, and soil‐enhancing species (Table 3; Figure 4) underscores the functional importance of these traits in successful shade tree integration.

**TABLE 3:** Convergence and divergence of farmers' local knowledge of the traits and ecological services of shade trees in comparison to published literature.

Local name	Botanic name	Ecological traits and services converging with published literature	Ecological traits and services diverging from published literature		Species uses
			Farmers LEK	MEK	
Oprono	*Mansonia altissima* A. Chev.	Fast growth, small crowns, and tall tree (Hawthorne 1995)	Evergreen	Highly deciduous (Hawthorne 1995)	Timber, fuelwood, woodworks
Asaa	*Chrysophyllum albidum* G.Don.	This species provides a lot of shade, and it can be used to make any wooden work (Orwa et al. 2009; Alowanou‐Kélé et al. 2023)			Fruits, medicinal, fuelwood, wood work
Awiemfuosemina	*Albizia ferruginea* (Guill. & Perr.) Benth.	Improves soil fertility, high adaptability, fast growth, windbreak, suppress weeds, control pest, shade, conserves moisture (Orwa et al. 2009)			Roofing
Cedrela	*Cedrela odorata* L.	No competition, adds nutrients to the soil, fast growing, suppress weeds, conserves soil moisture, reduces leaching, quality shade, deep roots, attracts beneficial insects, high adaptability, windbreak, reduces erosion, fairly drought and pest resistant (Piotto et al. 2004; Orwa et al. 2009)	Fast coppicing, N fixation	Non‐nitrogen fixing, not known for fast coppicing ability (Piotto et al. 2004; Orwa et al. 2009)	Timber, beautification, woodworks, boundary pillars, fuelwood, charcoal
Chenchen	*Antiaris toxicaria* (J.F.Gmel.) Lesch.	Fast growing, improves fertility, high adaptability (Hawthorne 1995; Orwa et al. 2009)			Timber, woodworks
Edinam	*Entandrophragma angolense* (Welw.) C.DC.	High adaptability, quality shade, prevent weeds, fast growth, conserves soil moisture, improves soil fertility, pest resistant, windbreak, prevents erosion, tall tree, small crowns (Hawthorne 1995; Orwa et al. 2009; Kyereh 2017)			Woodworks, timber, medicinal, boundary pillar, fuelwood
Emire	*Terminalia ivorensis* A.Chev.	Fast growing, fairly pest resistant, quality shade, reduces soil erosion, improves soil fertility, fast coppicing, suppress weeds, reduces leaching, conserves soil moisture, prevents mistletoes, host beneficial insects, drought resistant, small crowns, tall tree, improve soil aeration, deep rooted, supports soil organisms, moderate buttress, windbreak (Norgrove and Hauser 2002; Asare 2005; Asitoakor et al. 2024)	N fixing	Non‐nitrogen fixation	Wood works, timber, medicinal, fuelwood, mortar, fodder, charcoal, boundary pillars
Esa	*Celtis mildbraedii* Engl.	Fast growth, quality shade, reduces erosion, non‐host for pests and diseases (Orwa et al. 2009)			Medicinal, fuelwood, timber, local construction material
Mahagony	*Khaya grandifoliola* C.DC.	Windbreak, fast growth, conserves soil moisture, improves soil fertility, quality shade, reduces leaching, nutrient recycling, weed suppression, small buttress, attracts beneficial insects, non‐host for pests and diseases, survives harsh conditions, fast coppicing, supports soil organisms, drought resistant, microclimate amelioration, soil aeration, small crowns, tall tree (Lemmens 2008; Orwa et al. 2009)	N fixation	Non‐N fixing	Woodworks, timber, fodder, medicinal, charcoal, boundary pillars, beautification
Nkyene ne engo	*Cleistopholis patens* (Benth.) Engl. & Diels	Quality shade, reduces erosion, fast growth, non‐host for pests and diseases, tall tree, small crowns, evergreen (Hawthorne 1995)			Medicinal, fuelwood, timber, local construction material
Nyame dua	*Alstonia boonei* De Wild.	Fast growth, small crowns, tall tree, evergreen, quality shade, boost cocoa productivity, resists pests and diseases, reduces erosion, conserves soil moisture, attracts beneficial insects, improves fertility, survives harsh conditions, nutrient recycling, supports soil organisms (Asare 2005; Orwa et al. 2009; Asitoakor et al. 2024)			Medicinal, fuelwood, timber, local construction material, woodworks
Odum	*Milicia excelsa* (Welw.) C.C.Berg.	Improves soil fertility, conserves moisture, attracts beneficial insects, fast growth, reduces erosion, quality shade, nutrient recycling, reduces leaching, windbreak, weed suppression, soil aeration, tall tree, evergreen, fast coppicing (Orwa et al. 2009; Asitoakor et al. 2024)	Small crowns	Large crown (Orwa et al. 2009)	Woodworks, fuelwood, medicinal, timber, local construction materials, boundary pillars, molding
Ofram	*Terminalia superba* Engl. & Diels.	Fast growth, small crowns, tall tree, nutrients recycling, supports soil organisms, quality shade, windbreak, conserves soil moisture, reduces erosion, improves soil fertility, suppress weeds, attracts beneficial insects, deep roots, reduces nutrient leaching (Orwa et al. 2009; Kyereh 2017; Asitoakor et al. 2024)	Survives harsh conditions, prevents mistletoe	Not known for pest and diseases resistance, non‐N fixation (Orwa et al. 2009)	Woodworks, fuelwood, medicinal, timber, local construction materials, boundary pillars, molding, beautification, mortar
Ofuntum	*Funtumia elastica* (Preuss) Stapf	Quality shade (Asare 2005)			Woodworks, fuelwood, local construction material
Onyina	*Ceiba pentandra* (L.) Gaertn.	Fast growth, reduces erosion, conserves soil moisture, fast coppicing (Asare 2005; Harja et al. 2017)	Quality shade, non‐host for pest and diseases	Host for pest and diseases (Asitoakor et al. 2024)	Woodworks, timber, medicinal, fuelwood
Osese	*Holarrhena floribunda* (G.Don) T.Durand & Schinz	Evergreen, tall tree, small crowns, fast growth, quality shade, suppress weeds, nutrient recycling, improves soil fertility, reduce cocoa yields, damage to cocoa from falling branches, reduces soil erosion (Hawthorne 1995; Orwa et al. 2009)			Timber, medicinal, woodworks, local construction material, fuelwood, fodder
Sapele	*Entandrophragma cylindricum* (Sprague) Sprague	Fast growth, improve fertility, nutrient recycling, conserves soil moisture, suppress weeds, windbreak, quality shade (Asare 2005; Orwa et al. 2009)	Attracts beneficial insects		Woodworks, timber, local construction material, medicinal
Susumasa	*Newbouldia laevis* (P.Beauv.) Seem. ex Bureau	Quality shade (Orwa et al. 2009)			Medicine, fuelwood, woodwork
Watapuo	*Cola gigantea* A.Chev.	Quality shade (Orwa et al. 2009)			Medicinal, fuelwood, woodworks
Wawa	*Triplochiton scleroxylon* K. Schum	Quality shade, reduces erosion, fast growth, non‐host for pests and diseases (Orwa et al. 2009)			Medicinal, fuelwood, timber, woodworks, boundary pillar
Otie	*Pycnanthus angolensis* (Welw.) Warb.	Fast growing, improves fertility, high adaptability (Asare 2005; Asitoakor et al. 2024)			Timber, woodworks
Konkroma	*Morinda lucida* Benth	Fast growing, improves fertility, high adaptability (Kyereh 2017)			Local construction material, woodworks
Cassia	*Senna siamea* (Lam.) H.S. Irwin & Barneby	Fast growing, improves fertility, high adaptability (Nair et al. 1995)			Local construction material, woodworks

Abbreviations: LEK, local ecological knowledge; MEK, mainstream ecological knowledge.

Cluster two included species, such as *Albizia ferruginea, Antiaris toxicaria, Entandrophragma angolense, Entandrophragma cylindricum, Khaya grandifoliola, Terminalia ivorensis*, and *Terminalia superba* . Species in this cluster are predominantly fast‐growing and provide high‐quality shade, soil fertility enhancement, and erosion control. These traits are vital for maintaining soil health and preventing land degradation, which is a significant concern in cocoa‐growing regions (Asare et al. 2014; Kaba et al. 2020). *Albizia ferruginea* is highly adaptable and resistant to pests, which highlights the importance of resilience in shade tree selection. This is crucial for sustaining cocoa agroforestry systems, especially under changing climatic conditions (Appiah 2024; Boadi et al. 2024). Moreover, the preference for pest‐resistant species like *Terminalia ivorensis* indicates a strategic choice by farmers to minimize crop losses and reduce the need for chemical pesticides, thereby promoting sustainable farming practices (Asitoakor et al. 2024). Again, *Khaya grandifoliola* is noted for its fast coppicing, nutrient recycling, and pest resistance, making it a multifunctional species in agroforestry systems (Ngamau et al. 2004). The primary uses include timber, medicinal applications, and construction materials.

Cluster three was made up of four species namely, *Holarrhena floribunda, Morinda lucida, Mansonia altissima*, and *Cleistopholis patens* which are used for timber, medicinal purposes, and local construction materials and share traits like fast growth, adaptability, and the ability to suppress weeds (Hou et al. 2015). *Mansonia altissima* and *Morinda lucida* are examples of species with these characteristics, playing a significant role in both ecosystem services and economic benefits for farmers. Cluster four consisted of *Celtis mildbraedii, Triplochiton scleroxylon, Ceiba pentandra, Alstonia boonei*, and *Milicia excelsa*. Cluster four species were perceived to have robust ecological benefits, including reducing erosion, providing quality shade, and being non‐hosts for pests and diseases. They were highly valued by the farmers for their timber, medicinal properties, and use in local construction, reflecting their economic significance (Table 3). Furthermore, *M. excelsa* is recognized for its high‐quality timber and for improving soil fertility, moisture conservation, and nutrient recycling, which are critical for optimizing cocoa production (Smith Dumont et al. 2014; Nicaise et al. 2020). Thus, trees in cluster four may help maintain a stable environment, reducing temperature fluctuations and protecting cocoa plants from extreme conditions (Kyereh 2017; Nicaise et al. 2020).

### 3.5 Convergence and Divergence of Farmers' Local Knowledge of the Traits and Ecological Services of Shade Trees in Comparison to Published Literature

The study found a strong corroboration between farmers' LEK and scientific literature for 17 out of 23 preferred shade tree species, with partial alignment for the remaining six species (Table 3). This consistency reflects a well‐established understanding of the ecological roles and benefits of these trees, which is supported by scientific research (Anglaaere et al. 2011; Asare et al. 2014) and highlights the value of indigenous knowledge in understanding and managing shade trees in cocoa agroforestry systems (Asare et al. 2014). Convergence was seen in traits, such as fast growth, quality shade, and erosion control which reflects a shared recognition of the ecological traits and benefits of shade trees. However, divergence emerged regarding coppicing ability, crown architecture, and leaf phenology: 14 individuals erroneously attributed strong coppicing to *Cedrela odorata* , whereas the same number underestimated *Milicia excelsa* 's canopy size, and seven misclassified *Mansonia altissima* as evergreen. In contrast, misconceptions about nitrogen fixation, pest resistance, or non‐host status for cocoa pests were rare, with only two respondents expressing differing views. These divergent views highlight potential knowledge gaps, localized variations in tree performance under different environmental conditions or misinformation from extension officers (Personal Observation; Suvedi and Sasidhar 2024). The divergences reported in this study represent opportunities for co‐learning between farmers and researchers, which can enhance the adaptive capacity of cocoa agroforestry systems.

### 3.6 Priority Ecosystem Services Across Cocoa Production Stages

Provisioning services, such as food, fruits, and timber, were increasingly valued in older cocoa systems, peaking in very old cocoa farms (Table 4). This trend likely reflects the maturation of shade trees, which enhances their capacity to produce economically valuable outputs over time (Asare 2005; Asare et al. 2014). For example, shade trees can yield more fruits and timber when they mature which may explain the increased perceived value of provisioning benefits of shade trees in older agroforestry systems. Similarly, regulating and supporting services—such as weed suppression, soil moisture conservation, and erosion reduction—were consistently rated as important across all stages but gained heightened significance as cocoa farms aged. The increasing recognition of these services highlights' farmers' growing appreciation of the ecological functions that sustain cocoa productivity and resilience. For instance, the role of shade trees in improving soil health through nutrient recycling and erosion control is critical for maintaining long‐term agricultural sustainability (Asigbaase 2019; Fahad et al. 2022). These findings align with Bennett et al. (2009), who emphasized the multifunctionality of agroforestry systems in providing ecosystem services that support both ecological and economic stability.

**TABLE 4:** Mean rating score of ecosystem (dis)services across different cocoa production age stages. Likert scale ranged from 1 (lowest) to 5 (highest) (*n* = 363).

ES category	Specific services of shade trees	Cocoa production age‐stage mean rating score			
		YCF	MCF	OCF	VOCF
Provisioning	Food and fruits	3.6	4.3	3.9	4.1
	Timber and construction material	4.2	4.5	4.2	4.8
Regulating	Suppress weed growth	3.7	4.5	3.9	4.5
	Prolong the life of cocoa plantations	3.7	4.2	4.1	4.5
	Hosts pollinators	2.6	4.4	2.8	4.1
	Provide the required shade for cocoa	3.9	4.4	4.2	4.5
	Are not needed after cocoa systems mature	2.7	4.0	2.8	3.4
Supporting	Conserves soil moisture	3.4	4.4	3.9	4.7
	Reduces erosion	3.8	4.4	4.1	4.4
	Reduces nutrient leaching	3.8	4.3	4.0	4.4
	Supports soil organisms	3.7	3.9	3.8	3.8
Ecosystem disservices	**Specific disservices**				
	Reduce cocoa yields	3.7	3.7	3.8	3.5
	Competes for water and nutrients	3.9	3.9	3.8	4.0
	Competes for rooting space	3.8	3.7	3.9	3.9
	Cause damage to cocoa when their branches fall	4.0	4.2	3.9	4.4
	Hosts mistletoe	3.9	3.8	3.8	3.9
	Hosts mirids	3.7	3.9	3.8	3.8
	Hosts bad termites	3.9	3.8	3.8	3.7

Abbreviations: MCF, Mature cocoa farms; OCF, Old cocoa farms; VOCF, Very old cocoa farms; YCF, Young cocoa farms.

Despite the overall increase in perceived benefits, the necessity of shade trees in older cocoa systems elicited mixed perceptions among farmers. Although some continue to recognize their ecological and economic importance, others perceive them as less critical, potentially due to competition for resources (e.g., water, nutrients, rooting space) or risks associated with mature shade trees, such as falling branches or pest hosting (Lelamo 2021). These divergent views reflect the SES framework's recognition of trade‐offs and the dynamic feedback loops between ecological and social components.

Farmers identified several ecosystem disservices associated with shade trees, including competition for resources and compatibility issues. These disservices highlight ongoing challenges in managing shade trees in cocoa agroforestry systems. For instance, competition for water and nutrients may intensify as cocoa and shade trees mature, potentially constraining cocoa productivity. Similarly, risks from falling branches or shade trees hosting pests underscore the need for careful management to balance the benefits and trade‐offs of shade tree integration. Such challenges align with findings by Lelamo (2021), who reported similar issues in agroforestry systems. Within the SES framework, these disservices represent negative feedback loops that can influence farmers' decisions and management practices. Addressing these challenges requires adaptive strategies that integrate farmers' knowledge with ecological insights to optimize the benefits of shade trees while minimizing their drawbacks.

### 3.7 Drivers of Local Ecological Knowledge About Shade Trees on Cocoa Farms

The linear mixed‐effects regression results showed that production age of the cocoa farms significantly impacts LEK (Table 5). Farmers with matured cocoa farms had significantly higher LEK scores (mean = 93.87 ± 8.57) compared to young farms, with a positive slope of 3.03 (±1.44), indicating a significant increase (*p* = 0.04). However, farmers of old and very old cocoa farms showed an insignificant decrease in LEK relative to farmers of young cocoa farms. Farmers' LEK significantly increased with access to credit support, diverse FBOs membership, extension support, number of information sources, cocoa farming experience and income from cocoa, but it decreased with years of residence and number of farmlands. Thus, farmer socio‐economic and experience factors as well as information and support access factors shaped their LEK about shade trees. Access to financial resources would potentially enable farmers to invest in learning opportunities, such as training and workshops which may lead to greater LEK and that possibly explains their positive impact on LEK (Danso‐Abbeam and Baiyegunhi 2018; Avane 2019). Moreover, FBOs serve as platforms for sharing knowledge and experience among farmers which can enhance their understanding about shade trees and that may explain the positive impact of diverse FBOs membership on farmers' LEK (Ameyaw et al. 2018; Danso‐Abbeam and Baiyegunhi 2018). Again, access to multiple information sources or extension services can provide a broader perspective and updated knowledge on shade trees, which may contribute farmers' LEK (Joshi et al. 2004). Furthermore, the positive impact of cocoa farming experience on LEK reinforces the idea that experience over time contributes to better ecological understanding (Joshi et al. 2004; Ohmagari and Berkes 1997). Surprisingly, farmers' LEK decreased with the number of years of residence and farmlands which might be as a result of stagnation in learning or lack of new information sources, but further research is urgently needed to understand the learning dynamics of farmers (Winklerprins and Sandor 2003).

**TABLE 5:** Linear Mixed‐effects regression results of drivers of local ecological knowledge about shade trees on cocoa farms.

Parameter	Mean (SEM)	Slope/intercept (Beta)	df	Sig.
Intercept		81.58 ± 8.77	< 0.001	< 0.001
Productionage = matured	93.87 ± 8.57	3.03 ± 1.44	338.21	0.036
Productionage = old	90.48 ± 8.62	−0.36 ± 1.87	338.01	0.846
Productionage = very old	90.49 ± 8.71	−0.35 ± 2.37	338.09	0.882
Productionage = young	90.84 ± 8.63			
Residence years	41.19 (na)	−0.08 ± 0.03	338.01	0.020
Farmlands number	1.78 (na)	−0.99 ± 0.55	338.05	0.075
Farming years	25.34 (na)	0.09 ± 0.06	338.06	0.113
Credit access frequency	0.46 (na)	1.1 ± 0.32	338.02	0.001
Cocoa income	1130.51 (na)	1.45 × 10^−3^ ± 4.2 × 10^−4^	338.17	0.001
Number of information sources	2.13 (na)	1.04 ± 0.54	338.51	0.058
Extension support	3.09 (na)	0.4 ± 0.25	338.20	0.101
Membership of FBOs	3.29 (na)	1.9 ± 0.47	334.43	< 0.001

*Note:* FBO, farmer‐based organizations.

### 3.8 Perceived Opportunities and Barriers in Relation to Shade Tree Integration on Cocoa Farms

**FIGURE 5:**
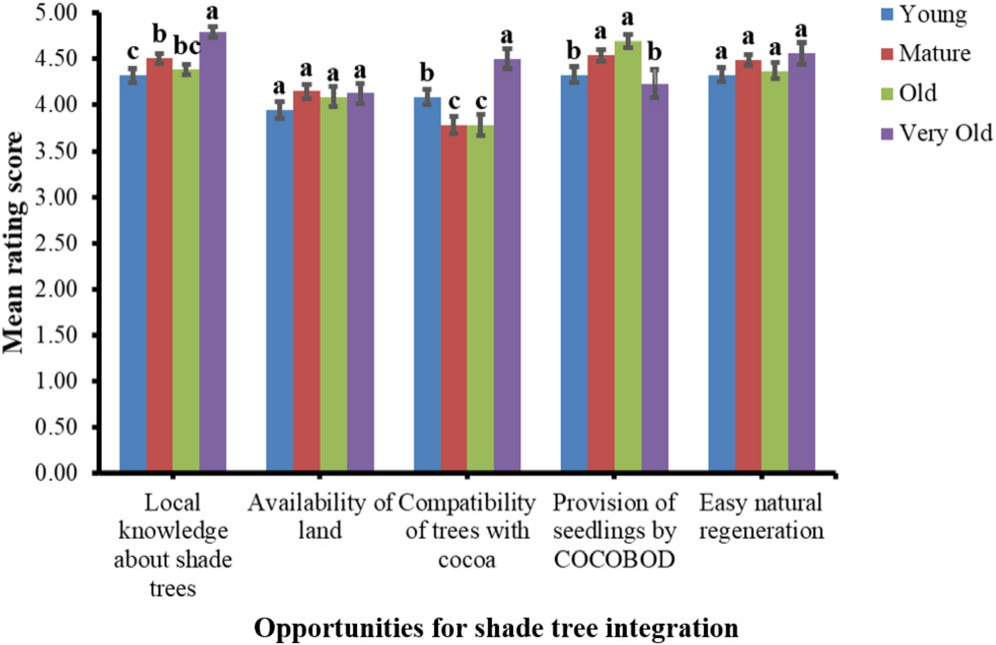
Perceived opportunities in relation to shade tree integration on cocoa farms. Mean rating scores indicate how important the perceived opportunities are across the cocoa production stages. Means that do not share a letter are significantly different.

Consistently across all cocoa production age stages, farmers considered easy regeneration, seedling supply by COCOBOD, shade tree compatibility with cocoa, land availability, and local ecological knowledge about their shades as opportunities for their integration on cocoa farms (Figure 5). More specifically, the rating for local knowledge about shade trees tended to increase from young to very old cocoa farms, which reflects its growing recognition and perceived role in sustaining shade tree populations over time (Asitoakor et al. 2023). Easy regeneration and local knowledge of shade trees can facilitate the establishment and maintenance of shade trees, reducing the need for external inputs and enhancing farm resilience (Mukhlis et al. 2022; Asitoakor et al. 2023). The rating of the provision of seedlings by COCOBOD (based on its free seedling provision program) as an opportunity for shade tree integration relatively increased across cocoa production age stages, peaking at OCF and declining to a mean score of 4.25 ± 0.14 in very old cocoa farms. This pattern suggests that while seedling supply is perceived as a critical factor for shade tree integration on cocoa farms, its relative importance may diminish as the farms mature due to increased potential for natural regeneration or reduced need for additional shade trees. The rating of land availability and easy natural regeneration as opportunities for shade tree integration was consistent across all cocoa production age stages, which suggests that adequate land resources are critical to enhancing shade tree integration on cocoa farms, regardless of farm age (Hoogendijk 2012). However, the higher rating of shade tree compatibility with cocoa as an opportunity for their integration by farmers of young and very old cocoa than farmers of mature and old cocoa suggests variability in how they perceive its relevance as the cocoa system ages.

**FIGURE 6:**
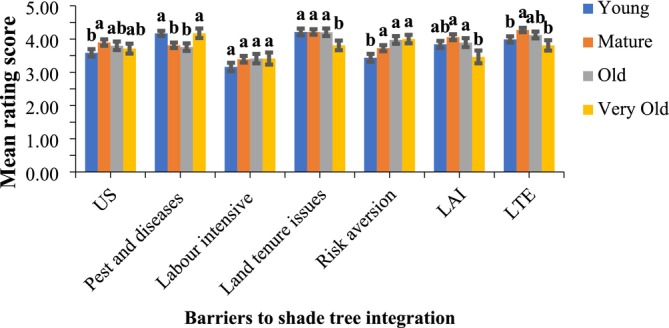
Perceived barriers in relation to shade tree integration on cocoa farms. Mean rating scores indicate how important the perceived barriers are across the cocoa production stages. Means that do not share a letter are significantly different. LAI = limited access to information; LTE = Limited training in relation to exotic trees; US = unavailability of seedlings.

A combination of knowledge gaps, resource constraints, and systemic issues were perceived as the key constraints to shade tree integration on cocoa farms (Wartenberg et al. 2018). More specifically, land tenure issues were the top barrier across young, mature, and old farms (mean ≥ 4.20) cocoa farms but significantly declined in very old cocoa farms (Figure 6). Limited training on exotic trees (LTE) and pest‐disease concerns also scored highly, peaking for LTE in mature farms (4.27). Limited access to information (LAI) was moderately important (highest in mature farms at 4.06, declining with plantation age), whereas risk aversion increased across cocoa ages. Conversely, labour intensity and seedling unavailability (US) ranked lowest overall, with US higher in mature farms and labour constraints remaining constant across all cocoa ages.

The prominence of land tenure issues underscores the necessity for policy reforms that clarify and secure land and tree tenure rights for cocoa farmers (Roth et al. 2017; Nasser et al. 2020). In Ghana, complexities surrounding tree tenure, including bureaucratic registration processes and limited awareness of tree rights, discourage farmers from integrating shade trees into their farms (Nasser et al. 2020). According to Duguma et al. (2019) and Carpente (2020), targeted training on tree species and their management is critical for successful agroforestry practices. Farmers may struggle to obtain relevant up‐to‐date information about shade tree management, which can affect their ability to make informed decisions and thus, emphasizes the need for improved extension services and knowledge dissemination (Duguma et al. 2001; Fleming et al. 2019; Carpente 2020). Asare et al. (2014) stressed the critical role of accessible information for effective shade tree management in cocoa systems. The emergence of pests and diseases as a barrier for young and very old cocoa farms indicates the changing nature of challenges as cocoa farms mature (Appiah 2024). Farmers of very old cocoa farms may be influenced by previous experiences or concerns about the impact of new shade trees on cocoa productivity. Interestingly, risk aversion strengthens with plantation age, suggesting that older stands' declining productivity induces caution toward new investments—an insight that management programs should address by offering rotational or intercropping models to demonstrate short‐term benefits of shade integration (Vaast and Somarriba 2014). Finally, the lower perceived significance of labour intensity and seedling unavailability (US)—despite being greater in mature farms—indicates that once tenure and knowledge constraints are reduced, practical resource issues may be more readily managed through community nurseries and labor‐sharing schemes (Kouassi et al. 2021).

## 4 Conclusions

This study contributes to our understanding of how farmers' shade tree preferences vary across cocoa production stages and how these preferences could influence both ecological resilience and conservation outcomes in cocoa agroforestry systems. Although farmers drew from a moderate (i.e., 23) pool of preferred species, their selection patterns shifted with the age of cocoa stands, indicating adaptive decision‐making that aligns with evolving ecological and management needs. The prioritization of functionally beneficial traits—such as fast growth, small crowns, and evergreen foliage—demonstrates farmers' capacity to balance productivity with ecological performance. Importantly, the finding that 40% to 57% of preferred species are under conservation threat underscores the dual role of shade tree integration in sustaining cocoa yields and conserving biodiversity. These results affirm the value of local ecological knowledge in shaping sustainable agroforestry practices and highlight opportunities for synergizing farmer‐driven strategies with formal conservation and extension programs. Experienced and financially supported farmers, including those in farmer‐based organizations with higher cocoa incomes, demonstrate better shade tree knowledge, emphasizing the role of experience and resources in ecological understanding. Our results offer critical insights into the socioecological logic guiding shade tree selection and provide a foundation for designing relevant, biodiversity‐friendly cocoa agroforestry interventions. We concluded that integrating preferred shade trees on cocoa farms has a potential conservation value; hence, conservation strategies should focus on protecting and propagating preferred shade tree species, especially those uniquely preferred at different cocoa growth stages. Additionally, the design of sustainable shade cocoa systems should prioritize shade trees which maximize both agricultural and ecological outcomes as the cocoa system evolves to meet farmers' preferences. Future research should incorporate longitudinal ecological assessments to extend our results.

## Author Contributions

**Michael Asigbaase:** conceptualization (lead), data curation (lead), formal analysis (lead), investigation (lead), methodology (lead), project administration (lead), resources (lead), supervision (lead), validation (lead), visualization (lead), writing – original draft (lead), writing – review and editing (lead). **Solomon Batamia Ndego:** data curation (supporting), resources (supporting), validation (supporting), visualization (supporting), writing – original draft (supporting). **Belinda Effah:** formal analysis (supporting), project administration (supporting), validation (supporting), visualization (supporting), writing – original draft (supporting).

## Ethics Statement

The study complied with both local (Ethics Committee of the Department of Forest Science, University of Energy and Natural Resources) and international (guidelines Helsinki Declaration of 1975, revised in 2000).

## Consent

All participants of the research informed of the purpose and aims of the search and they consented to they were engaged. Consent to publish: All farmers who participated in the research were informed of the intention to publish the results of the research, and they consented to it.

## Conflicts of Interest

The authors declare no conflicts of interest.

## Supporting information


Appendix S1.

Appendix S2.

Appendix S3.

## Data Availability

All relevant data are included within the main manuscript and the accompanying [Link], [Link], [Link] files.
