# Supplementary material for: Shade Tree Selection in Cocoa Agroforestry: Ghanaian Farmers' Preferences, Ecological Insight and Drivers of Local Ecological Knowledge
Source: Ecol Evol. 2025 Jun 30;15(7):e71685. doi: 10.1002/ece3.71685 (PMC12209332; doi:10.1002/ece3.71685)
Supplement: Supplementary file 1 — Appendix S1. [file ECE3-15-e71685-s003.docx]

**Supplementary information 1: Socio-economic characteristics of cocoa farmers across different cocoa production stages.**

| **Category** | **Variable** | **YCF** | **MCF** | **OCF** | **VOCF** | **X^2^-value/F-Value** | **P-value** |
| --- | --- | --- | --- | --- | --- | --- | --- |
| **Frequency** | | | | | | |  |
| Gender | Female | 33 | 36 | 32 | 16 | 7.292 | < 0.001 |
|  | Male | 65 | 106 | 42 | 32 |  |  |
| Marital status | Single | 8 | 8 | 3 | 3 | 12.108 | < 0.001 |
|  | Married | 66 | 75 | 40 | 35 |  |  |
|  | Widowed | 6 | 3 | 10 | 6 |  |  |
|  | Divorced | 4 | 5 | 2 | 4 |  |  |
| Nativity | Migrant | 24 | 23 | 8 | 17 | 6.207 | < 0.001 |
|  | Native | 61 | 67 | 47 | 31 |  |  |
| Religion | Christian | 86 | 121 | 70 | 42 | 6.415 | < 0.001 |
|  | Muslim | 9 | 12 | 1 | 4 |  |  |
|  | Traditional | 3 | 9 | 3 | 2 |  |  |
| Educational level | No formal education | 7 | 22 | 10 | 11 | 18.069 | < 0.001 |
|  | Primary | 3 | 14 | 5 | 1 |  |  |
|  | JHS | 28 | 46 | 20 | 11 |  |  |
|  | SHS | 49 | 55 | 34 | 23 |  |  |
|  | Tertiary | 9 | 6 | 4 | 2 |  |  |
| **Mean ± SEM** | | | | | | |  |
|  | Household size | 5.56 ± 0.29 | 6.22 ± 0.25 | 6.85 ± 0.38 | 6.88 ± 0.58 | 3.032 | 0.029 |
|  | Residence years | 33.06 ± 1.8 | 38.53 ± 1.24 | 51.61 ± 2.14 | 49.53 ± 2.89 | 21.378 | < 0.001 |
|  | Respondent’s age (years) | 44.52 ± 1.36 | 51.69 ± 0.94 | 61.59 ± 1.33 | 61.67 ± 2 | 36.169 | < 0.001 |
|  | Household adults | 3.47 ± 0.18 | 3.89 ± 0.18 | 4.65 ± 0.28 | 4.92 ± 0.42 | 6.642 | < 0.001 |
|  | Household children | 2.09 ± 0.18 | 2.41 ± 0.15 | 2.25 ± 0.19 | 1.88 ± 0.26 | 1.357 | 0.256 |
|  | Number of farmlands | 1.43 ± 0.07 | 1.76 ± 0.07 | 2.11 ± 0.13 | 1.96 ± 0.14 | 8.514 | < 0.001 |
|  | Total farmlands size (ha) | 3.89 ± 0.31 | 5.91 ± 0.56 | 6.63 ± 0.61 | 5.75 ± 0.53 | 4.044 | 0.008 |
|  | Cocoa farming years | 10.51 ± 0.58 | 24.42 ± 0.56 | 34.51 ± 1.37 | 42.08 ± 2.19 | 157.671 | < 0.001 |
|  | Credit access frequency | 0.36 ± 0.12 | 0.39 ± 0.09 | 0.36 ± 0.13 | 0.96 ± 0.44 | 2.086 | 0.102 |
|  | Monthly cocoa income (US$) | 563.51 ± 91.16 | 1433.01 ± 145.74 | 1253.32 ± 122.14 | 1208.54 ± 130.12 | 8.423 | < 0.001 |
|  | Monthly non-cocoa income (US$) | 777.97 ± 144.25 | 575.52 ± 82.1 | 508.91 ± 113.93 | 399.78 ± 85.32 | 1.606 | 0.188 |
|  | Extension (2022) | 2.35 ± 0.19 | 3.18 ± 0.2 | 3.55 ± 0.18 | 3.33 ± 0.26 | 5.804 | 0.001 |
|  | Extension (5yrs ago) | 6.12 ± 0.75 | 11.84 ± 0.89 | 13.22 ± 0.94 | 15.19 ± 1.49 | 15.973 | < 0.001 |
|  | Membership of farmers organizations | 3.4 ± 0.17 | 3.04 ± 0.15 | 3.21 ± 0.17 | 3.98 ± 0.05 | 4.821 | 0.003 |
|  | Number of information sources | 1.79 ± 0.09 | 2.23 ± 0.09 | 2.16 ± 0.1 | 2.37 ± 0.15 | 5.406 | 0.001 |

Note US$ 1 = GH₵ 9.91 (Inter-Bank Exchange Rate – 2023 January Month Average, Bank of Ghana). YCF = Young cocoa farms, MCF = Mature cocoa farms, OCF = Old cocoa farms, VOCF = Very old cocoa farms.
