# Supplementary material for: Shade Tree Selection in Cocoa Agroforestry: Ghanaian Farmers' Preferences, Ecological Insight and Drivers of Local Ecological Knowledge
Source: Ecol Evol. 2025 Jun 30;15(7):e71685. doi: 10.1002/ece3.71685 (PMC12209332; doi:10.1002/ece3.71685)
Supplement: Supplementary file 2 — Appendix S2. [file ECE3-15-e71685-s001.docx]

**UNIVERSITY OF ENERGY AND NATURAL RESOURCES, SUNYANI, GHANA**

**SCHOOL OF NATURAL RESOURCES**

**DEPARTMENT OF FOREST SCIENCE**

*Dear Respondents*

*This questionnaire is administered to solicit information related to trees cocoa farmers prefer on their farms as the cocoa trees mature. Your responses will provide data on (i) demographic, economic and farm characteristics, (ii) shade tree preferences across cocoa production stages, (iii) farmers' perceptions of ecosystem services and disservices, and (iv) barriers and opportunities for shade tree integration. The outcome of the research will be a manuscript that will be published to advance current knowledge on shade tree integration on cocoa farms. Your candid answers and opinions are therefore solicited. Be assured that your responses would be treated with the utmost confidentiality and your personality anonymized. Thank you.*

**A. Socio-demographic characteristics**

1. Respondent’s ID……..……………………………………………………………………………….

2. Name of district ……………………………………………………………………………………….

3. Name of community………………………………………………………………………………….

4. Gender/Sex………………………………………………………………………………………………

5. Marital status:

i) Single [ ]

ii) Married [ ]

iii) Widowed [ ]

iv) Divorced [ ]

6. Age ………………………………………………………………………..

7. Household size of respondent……………………………….

8. Number of children (younger than 18 years) in the household of respondent.....................

9. Number of adult members (18years or older) in the household of respondent...................

10. Number of years of residing in the community...................................................................

11. Ethnicity of respondent…………………………………………….

12. Religion:

i) Christian [ ]

ii) Muslim [ ]

iii) Traditionalist [ ]

iv) Other (please specify)…………………………………….

13. Highest educational level:

i) Primary [ ]

ii) JHS [ ]

iii) SHS/TEC/VOC [ ]

iv) Tertiary [ ]

v) No formal education [ ]

vi) Other (please specify)……………………………..

14. Origin of respondent:

i) Native [ ]

ii) Immigrant [ ]

15. How long have you been a cocoa farmer (years)?.....................

16. How many farmer-based organizations are you a member of?...........

17. Mention all the farmer-based organization you belong to…………………

**B. Farm characteristics**

18. How many farmlands do you have?..........................................................

19. What is the total size of all your farmlands?.............................................

20. What is the nature of land ownership of your farmland?.........................

21. How old are your cocoa trees (over 80% of cocoa trees on the farm)?.............

22. What is your average cocoa yield during the major season (bags)?............................

23. What is your average cocoa yield during the minor season (bags)?…………………………

24. How many times have you received extension services last year?

25. How many times have you received extension services during the past five years?

**C. Economic attributes**

26. What is your main source of income?

27. Mention all other sources of income to you.

28. Did you obtain credit/loan for cocoa production this year?

29. How many times have you obtained credit/loan for cocoa farming over the past 5 years

30. What is your average monthly cocoa income?

31. What is your average monthly off-farm income?

**D. Farmers’ perception of shade trees and priority ecosystem services**

32. Mention all your sources of information about shade trees……………..

33. Priority ecosystem services of shade trees

| **Ecosystem (dis)services of shade trees** | **Rating score [0(lowest) to 5 (Highest)]** |
| --- | --- |
| i. Conserves soil moisture |  |
| ii. Reduces erosion |  |
| iii. Reduces nutrient leaching |  |
| iv. Supress weed growth |  |
| v. Prolongs the life of cocoa plantations |  |
| vi. Provides food and fruits |  |
| vii. Provides timber and construction material |  |
| viii. Host pollinators |  |
| ix. Provides quality shade for cocoa |  |
| x. Shade trees are not needed after cocoa systems mature |  |
| xi. Supports soil organisms |  |
| xii. Competes for water and nutrients |  |
| xiv. Competes for rooting space |  |
| xv. Causes damage to cocoa when their branches fall |  |
| xvi. Hosts mistletoe |  |
| xvii. Hosts mirids |  |
| xviii. Reduces cocoa yields |  |
| xix. Hosts bad termites |  |

**E. Preference and local ecological knowledge of shade trees among cocoa farmers**

| **Sn.** | **Species name** | **Reasons (ecological traits and services)** | **Uses** |
| --- | --- | --- | --- |
| 1 |  |  |  |
| 2 |  |  |  |
| 3 |  |  |  |
| 4 |  |  |  |
| 5 |  |  |  |
| 6 |  |  |  |

**F. Barriers, opportunities, and strategies for shade tree integration**

**34. Barriers to shade tree integration on cocoa farms**

| **Barriers** | **Rating score [1 (lowest) to 5 (highest)]** |
| --- | --- |
| i. Unavailability of seedlings |  |
| ii. Pest and diseases |  |
| iii. Labour intensive |  |
| iv. Land tenure issues |  |
| v. Not wanting to take risk |  |
| vi. Limited access to information |  |
| vii. Limited training in relation to exotic trees |  |

35. What other constrains prevents you from integrating trees on cocoa farms (mention all?

**36. Opportunities for the integration of shade trees on cocoa farms**

| **Opportunities** | **Rating score [1 (lowest) to 5 (highest)]** |
| --- | --- |
| i. Local knowledge about trees |  |
| ii. Availability of land |  |
| iii. Compatibility of trees with cocoa |  |
| iv. Provision of seedlings by COCOBOD |  |
| v. Easy natural regeneration |  |

37. What other opportunities are there for the integration of shade trees on cocoa farms (mention all).

**“Shade Tree Selection in Cocoa Agroforestry: Ghanaian Farmers’ Preferences, Ecological Insight and Drivers of Local Ecological Knowledge”**

**Information Sheet for Participants**

**Invitation**

You are being invited to be involved in a research study; before you decide whether you want to take part, it is important for you to understand why the research is being done and what your participation will involve. Please take time to listen to the following information carefully and discuss it with other people if you wish. Please contact me if anything is unclear or if you would like more information. Take time to decide whether or not you wish to take part. The study complies with the University of Energy and Natural Resources Ethics Committee’s requirements.

**What is the purpose of this study?**

The aim of this study is to document and analyze shade tree species cocoa farmers prefer as their cocoa trees mature. It aims to contribute in the conservation of tree species and enhance cocoa production. The study is purely for academic purposes (i.e. it is non-commercial).

**Why I have been chosen?**

You are randomly selected because you live in this community. There is no specific reason other than that you are one of those who are resident in this community to whom the study is relevant.

**What will participation involve?**

You will be asked a number of questions relating to: (i) demographic and farm characteristics, (ii) shade trees you prefer, (iii) farmers' perceptions of ecosystem services and disservices, and (iv) barriers and opportunities for shade tree integration. Your responses to the questions will be recorded and processed together with responses from others to achieve the purpose of this research. We will visit your farm together with you to identify and confirm the species you mentioned.

**What if I decide that I don’t want to take part?**

You are free to decide that you don’t want to take part in the study and can:

1. Refuse to answer any questions that you don’t want to
2. Decide to stop the interview at any time
3. Remove your consent for the data collected to be used.

**Will I be paid for my time?**

There is no payment for taking part in this study. However, research findings would be shared with the participants through a workshop or a presentation depending on the availability of resources.

**Will I be anonymous, and who will know my identity?**

If you agree to take part in an interview, a Participant ID Number will be generated for you, and that’s the only thing that will be used to identify you. Your identity will only be known by the interviewer, and will not be found in any record. Hard copy and electronic data will be stored on the University of Energy and Natural Resources computer system and it this will be deleted after 4 years, or if you withdraw your consent (whichever is sooner).

**What do I do next?**

If you’d like to be involved in this study, we will interview you right away or when we visit you at your home community centre or on your farm (whichever is suitable for you). Before the interview you’ll be asked to provide an oral consent.

**Who shall I contact with any questions?**

To be part of this study, or to ask any questions, then please get in contact with the Principal Investigator, Dr Asigbaase Michael:

Email: michael.asigbaase@uenr.edu.gh

Tel.: 0508168737.
